# Low-level sensory processes play a more crucial role than high-level cognitive ones in the size-weight illusion

**DOI:** 10.1371/journal.pone.0222564

**Published:** 2019-09-13

**Authors:** Cody G. Freeman, Elizabeth J. Saccone, Philippe A. Chouinard

**Affiliations:** Department of Psychology and Counselling, School of Psychology and Public Health, La Trobe University, Melbourne, Victoria, Australia; University of Exeter, UNITED KINGDOM

## Abstract

The size-weight illusion (SWI) pertains to the experience of perceiving the smaller of two equally weighted objects as heavier. Competing theories to explain the illusion can be generally grouped into cognitive and sensory theories, which place more importance on top-down processing of cognitive expectations and bottom-up processing of sensory information about the size and weight of objects, respectively. The current study examined the relative contribution of these two general explanations. This was done by varying the amounts of cognitive load in a dual-task and the quality of somatosensory feedback by wearing or not wearing gloves. Participants placed their hands through a curtain inside a box so they could not see the test objects. Inside the box, they were presented with either a small or large sphere of varying weights, which they explored manually without vision. Participants provided magnitude estimates about each object’s weight in four experimental conditions (no-load with gloves, no-load without gloves, low-load without gloves, and high-load without gloves). The dual-task involved the visual presentation of a cross on a computer monitor that changed in both colour and orientation. With foot pedals, the participants responded to a target colour and / or orientation, which varied across conditions, while they hefted an object. Some conditions were designed to be more cognitively taxing than others (high-load > low-load > no-load conditions). The results revealed that the strength of the SWI diminished when participants wore the gloves but did not change as cognitive load increased on the dual-task. We conclude that the illusion is more influenced by bottom-up sensory than top-down cognitive processes.

## Introduction

The size-weight illusion (SWI) is a prominent weight illusion that occurs when handling objects of different sizes but equal mass [1]. Interacting with these objects causes an individual to perceive the smaller object as heavier than the larger one when they have the same weight. The SWI has been reported to be cognitively impenetrable [2–4]. Namely, participants will continue to experience the illusory difference in weight with repeated exposure and full knowledge that they have the same weight [4, 5]. Various researchers have attempted to comprehend the mechanisms driving the SWI [6, 7, 8] but a unifying theory has yet to be established [2, 9]. However, most theories on the SWI can be sorted generally into either cognitive or sensory explanations.

Cognitive accounts place more importance on the influence of previous knowledge [2] and the application of learned expectations of weight [10–12]. To explain how these expectations are formed, one should consider the relationship between size and weight in the real world. The natural size-weight relationship is positively correlated with larger objects typically weighing more than smaller ones. This creates the learned association that larger objects weigh more than smaller ones [6, 13]. However, the equally weighted size-weight objects violate these expectations [2, 14]. When a person lifts the small and large objects in the SWI, they expect the former to be light and the latter to be heavy. Some researchers believe that this mismatch between expectation and actual weight causes people to perceive their weights differently [15]. Namely, people discover while lifting the objects that the smaller one is heavier than expected and requires more force to be lifted, making that object feel heavier, while the larger one is lighter than expected and requires less force to be lifted, making that object feel lighter. Evidence suggests that the perception of the illusion may not depend on the force used to lift the objects [4].

Research surrounding cognitive accounts of the illusion has allowed for some creative experiments. For example, Ellis and Lederman [12] demonstrated a weight illusion in golfers but not in non-golfers using real and practice golf balls. This was achieved by altering the two balls to be equal in weight. Under these circumstances, only golfers possessed the knowledge, and therefore expectation, that practice golf balls are lighter than the real ones. As a result, the golfers experienced the practice golf ball as heavier while the non-golfers did not. A further example has been reported in equally weighted children’s dolls representing different genders, whereby female dolls feel heavier than male ones [11, 16]. Another example comes from Buckingham and Goodale [10] in which the authors led their participants to believe that they were lifting different objects varying in size when they were in fact lifting the same object consistently on every trial. Participants experienced the object as being heavier when they were led to believe that the object was smaller. Conversely, the participants experienced the object as being lighter when they were led to believe that the object was larger.

In contrast, sensory accounts of the SWI place more importance on low-level stages of sensory processing [17, 18]. One example of a sensory theory proposes that density processing influences people’s conscious perception of weight [17–19]. To explain, SWI objects differ in size but not weight and their density will therefore be higher for the smaller object than the larger one–given that density is derived from an object’s weight divided by its volume. To process density, the brain needs to gauge both an object’s size as well as its weight. It is theorised that weight and density enter consciousness as if they were similar things–much like we sometimes mistake flavour for taste, such as the perception of a jalepeno pepper being ‘hot’ [20]. The jalepeno’s temperature is not different from other foods. Rather, the sensation of hotness is created from molecules in the jalepeno interacting with the nociceptors in the mouth and nose that are normally triggered by pain and higher temperatures.The merits of sensory accounts have been tested by comparing differences in the SWI when participants held the objects directly in the palm of the hand [21, 22] and when they lifted them using a pulley system or strings [21, 23]. This is because these different ways of lifting objects change the type of sensory information that is gathered and processed about their size. If sensation plays an important role then there could be differences in how the illusion is experienced when size is processed by different sensory channels–in the same way a jalepeno pepper feels excruciatingly more painful if it touches the eye than when it enters the mouth [20]. The manual exploration of objects allows touch receptors on the skin to gather size information haptically and proprioceptive receptors in the muscle spindles to gather size information kinaesthetically from sensing torques applied on the objects during movement. Conversely, lifting objects using either a pulley system or strings removes this somatosensory (i.e. both haptic and kinaesthetic) information about their size–leaving only vision to process size.

Ellis and Lederman [21] used this approach to alter the availability of somatosensory and visual information about object size in the SWI. In one condition, participants were blindfolded and obtained only somatosensory information about object size through manual exploration. In a second condition, participants were not blindfolded and obtained only visual information about the size of objects by lifting them using strings. In a third condition, participants could see the objects during manual exploration, allowing both somatosensory and visual information about their size to be be processed. The authors demonstrated that the strength of the SWI was dependent on sensory channels. The illusion was stronger when somatosensory information about size was available and that the availability of visual information about size made no difference to illusory strength when somatosensory information about size was present.

The importance of somatosensory feedback in the SWI has garnered further support from the case study of patient IW who suffers from peripheral deafferentation, a condition in which one can no longer process proprioceptive or tactile input [24]. Instead, patient IW must rely on visual feedback about his body and how it interacts with objects to process their size and weight [24]. Remarkably, patient IW’s use of this compensatory strategy allows him to lift objects and judge their weight to a degree that is as accurate and proficient as age-matched controls [25]. Additionally, when presented with SWI objects, patient IW’s pre-lift expectations mirror those of the typical relationship between size and weight seen in neurologically intact individuals. Specifically, he expects the largest of three objects to be heavier than the medium one and the medium one to be heavier than the smallest one. However, when lifting the SWI objects, he does not experience the illusion at all–demonstrating that somatosensory feedback about their size and weight are necessary for the perceptual illusion to occur. Given that a total absence of somatosensory information causes the lack of an illusion, one could also expect that simply lowering the quality of somatosensory information would lessen the perceptual magnitude of the illusion rather than eliminating it altogether. One potential way to alter the quality of somatosensory information would be to wear thick winter gloves. Doing so should allow us to test how simply lowering the quality of haptic information rather than removing it might impact the perceived strength of the SWI.

On balance, previous research seems to support both top-down cognitive and bottom-up sensory accounts of the SWI. Evidence supporting a more crucial role for the processing of object size by somatosensory than visual channels undermines top-down accounts of the illusion yet there is still clear evidence that expectations influence weight perception. Thus, there are merits to both accounts. The question remains to what degree the two contributes to the SWI. The present investigation attempts to shed light to this question by varying the cognitive load on a secondary task and the quality of somatosensory feedback about the size and weight of objects. Both top-down cognitive and bottom-up sensory accounts of the SWI make different predictions. Top-down cognitive accounts predict that the SWI decreases with cognitive load on a secondary task while bottom-up sensory accounts predict that the SWI dimishes with the quality of somatosensory feedback about the size and weight of objects.

For the purposes of this study, cognitive load is defined as the amount of mental effort devoted to processing multiple pieces of information for the purposes of understanding and directing behaviour. The underlying basis of many cognitive accounts is that the illusion is driven by the application of previous knowledge to sensory input, which requires some level of cognitive load [26, 27]. Individuals have limited cognitive load capacity [28, 29] and therefore a processing bottleneck typically occurs for non-automatised operations on a dual-task [30]. As a result, if a primary task requires cognitive load then performing a cognitively demanding secondary task should interfere with the primary task [26]. In the context of the SWI, performing a secondary task should interfere with the perceptual strength of the illusion as a function of the degree to which cognitive processing is important in driving the illusion. That is, if the illusion relies strongly on the application of prior cognitive expectations to the lifted objects, then taxing these cognitive resources with another task should in theory reduce the magnitude of the illusion [31]. On the other hand, the strength of the illusion should remain largely unaffected by a secondary task if it is driven more strongly by automatic low-level sensory mechanisms [26, 32].

As far as we know, only two SWI studies have incorporated a dual-task. In one, Baugh et al. [33] had participants in a separate control experiment move objects to different locations based on their colour to determine whether fingertip forces applied during lifting would be affected. Although this additional requirement interfered with how the objects were lifted, the authors did not report how this may have also affected weight perception. In the second, Trewartha and Flanagan [34] had participants complete a mental arithmetic task whilst lifting SWI objects. The participants were audibly presented with a number between 12 and 99, which they had to mentally subtract seven and vocalise their response as quickly as possible. No effects on the dual-task were observed on weight perception. However, the focus of the authors’ study was to determine if attention was required in the updating of priors in the SWI using other manipulations. The dual task as a manipulation was of secondary importance to them. In both studies, not much detail was provided about the results obtained in the secondary tasks nor was there much discussion about them. Given the paucity of information provided, it is unclear how cognitively taxing participants found the secondary tasks. Both studies leave many unanswered questions regarding the impact of cognitive load on the strength of the SWI.

In addition, we investigated the contribution of sensory mechanisms on the SWI by diminishing the quality of somatosensory feedback about the size and weight of the objects. Specifically, we employed a condition during which participants could not see the SWI objects but hefted them while wearing fleece gloves. The gloves diminished somatosensory information regarding their size and weight. As seen in the study of patient IW, a total absence of somatosensory information causes the lack of an illusion [24]. Therefore, one could also expect that simply lowering the quality of somatosensory information would lessen the perceptual magnitude of the illusion rather than eliminating it altogether. This allows us to investigate whether reducing the quality of somatosensory feedback, in the absence of visual access, has an impact on the perceived strength of the illusion. We reasoned that wearing gloves should interfere with the perceptual strength of the illusion as a function of the degree to which low-level sensory mechanisms are important in driving the illusion. Surprisingly, this manipulation on the SWI has never been carried out before even though there appears to be a general assumption that wearing gloves would reduce the strength of the illusion. The present investigation is the first to test this possibility empirically.

## Materials and methods

### Overview

The study began with the completion of a series of pre-experiment questionnaires and vision checks. The vision checks were necessary to verify that participants could differentiate between visual stimuli in the secondary task. The remainder of the experiment consisted of a familiarisation phase, followed by four experimental conditions. The four conditions consisted of no-load with gloves, no-load without gloves (hereafter called the ‘no-load’ condition), low-load without gloves (hereafter called the ‘low-load’ condition), and high-load without gloves (hereafter called the ‘high-load’ condition). All participants completed all conditions in a session lasting approximately 90 minutes. The familiarisation phase allowed participants to get comfortable with the experimental set-up and hefting procedures. Following this, there were four experimental conditions completed in an order counterbalanced across participants by a Latin square. Each condition included a hefting task whereby participants used one of their hands to heft a plastic sphere. This task was identical for each of the experimental conditions. The low-load and high-load conditions included a secondary task as well as the hefting task. Participants did not complete a secondary task while hefting an object in the no-load conditions. The no-load with gloves condition had participants wear fleece gloves. Participants provided perceptual magnitude estimates of weight following each trial, which were then transformed into a z-score.

### Participants

Twenty-five participants (13 males, all right-handed) ranging between 18 and 36 years of age (*M* = 22.80, *SD* = 3.83) with normal or corrected-to-normal vision took part in the experiment. Participant handedness was verified using the Edinburgh Handedness Inventory [35]. Visual acuity was verified using the Snellen Chart [36] with all participants obtaining a score of at least 20/40 on their better eye. Participants were checked for colour vision deficiencies with the Ishihara colour plates [37]. All participants met our screening criteria and moved on to the experimental apparatus to begin the next phase of the study. Participants provided informed written consent and received a gift voucher for their time. This study was approved by La Trobe University’s Human Ethics Committee. The individual in this manuscript has given written informed consent (as outlined in PLOS consent form) to publish their picture in one of the figures.

### Experimental apparatus and stimuli

**Fig 1:**
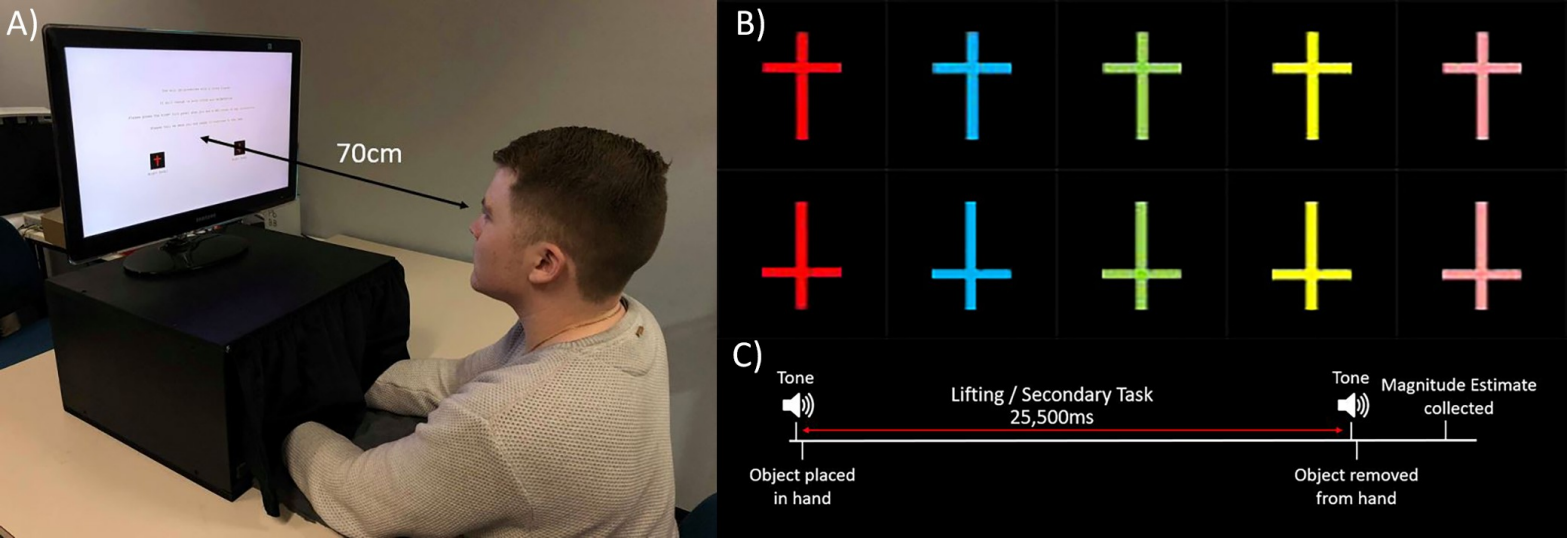
Experimental apparatus, stimuli, and trial sequence. (A) Experimental set-up used for the experimental procedures. The box was attached with a curtain so that participants could not see the object that they were hefting. (B) Upright and inverted versions of the five coloured crosses used in the secondary task. (C) Diagram of sequence of events in an experimental trial.

The following set-up was used (Fig 1). Participants placed both hands inside a wooden box (50cm wide, 28cm high, and 45cm deep) with a curtain attached to ensure they could not view the object that they were handling. On top of the box was a 23-inch Samsung LCD monitor (1920 x 1080 pixel resolution, 60 Hz frame rate), which presented stimuli for the secondary task using E-Prime 2.0 software [38]. The position of the monitor was fixed at a viewing distance of 70 cm. Because the participants’ hands were used for the hefting task, they used their feet to make responses in the secondary task via a set of Treadlite II foot-pedals instead (Linemaster Switch Corporation; Woodstock, Connecticut, USA). These foot-pedals were connected to a Chronos response box [39] to collect response accuracy and reaction time (RT).

**Fig 2:**
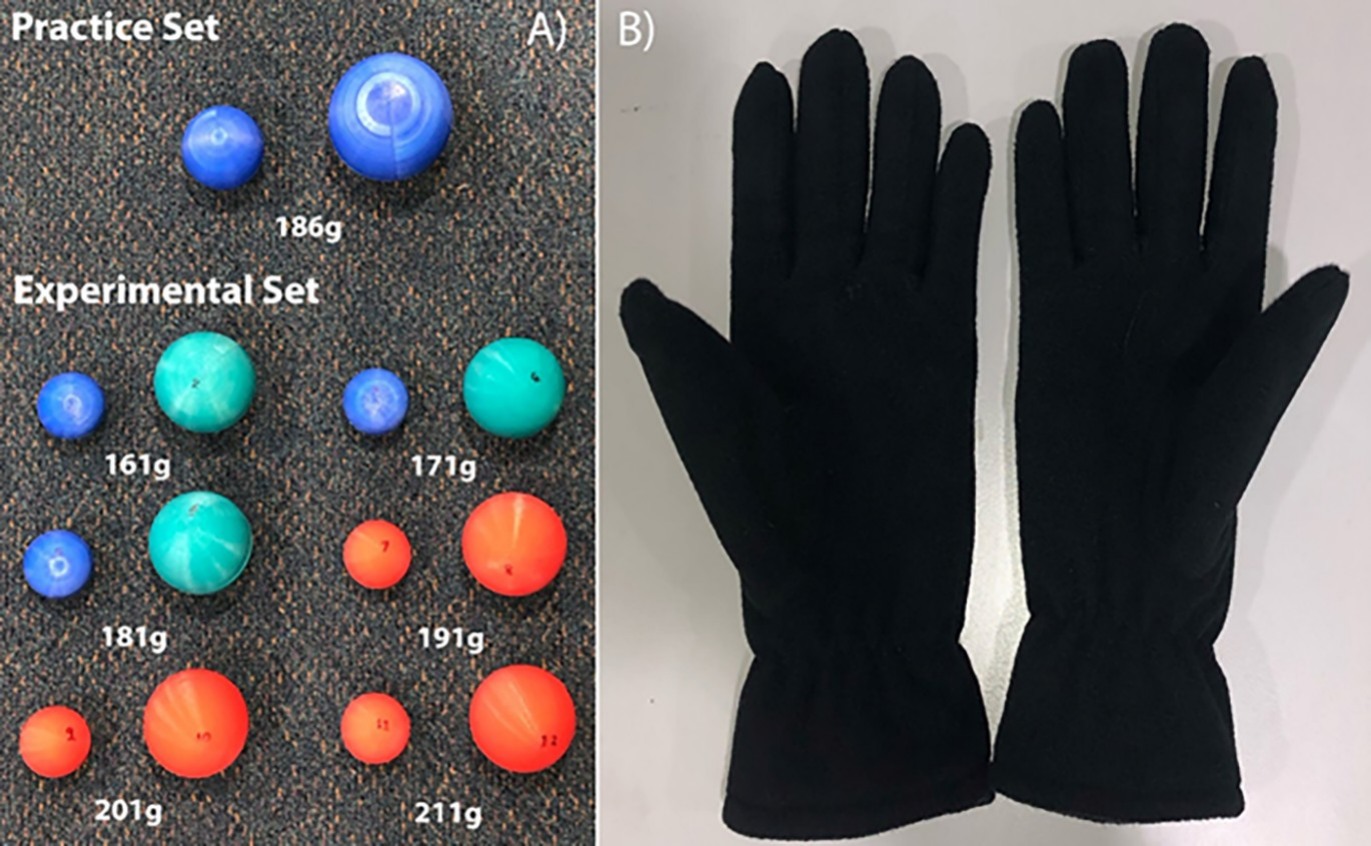
Practice and experimental stimuli and gloves. (A) Stimuli used for the hefting task. Spheres were printed from PLA plastic and weighted with lead pellets and packing to maintain normal centre of gravity. The spheres were presented inside the apparatus box and as such were never seen by the participants. Thus, the different colours of the stimuli shown in this figure had no effect on the participant’s performance. (B) Fleece gloves used to reduce the quality of somatosensory information in the no-load with gloves condition. Each glove weighed 36 g and all participants wore the same pair of gloves.

Stimuli for the hefting task were seven pairs of plastic spheres varying in weight between 161 g and 211 g in 10 g increments (Fig 2). The spheres were 3D printed from polylactic acid (PLA) plastic using a BQ Witbox 2 printer (BQ; Las Rozas, Madrid, Spain). Each sphere was then weighted with varying amounts of lead pellets to ensure equal weight between pairs. These lead pellets were distributed in the centre of the sphere with padding to maintain a regular centre of gravity. The spheres were split into two sets: a set of practice stimuli and a set of experimental stimuli. The practice set consisted of a single pair and was used for the familiarisation phase only. They measured 60 mm and 90 mm in diameter and both weighed 186 g. The experimental set was made up of six pairs that were used for the experimental conditions. These pairs were all 42 mm and 62 mm in diameter.

### Hefting (primary) task

The hefting task required the participants to place their hands inside a box through a curtain. An object was placed onto one of their hands by the experimenter and they were instructed to heft the object for the duration of each trial, at which point it was removed from their hand by the experimenter. The task was performed in each hand to reduce fatigue and make it more engaging. We did not have any specific hypothesis with regards to how the SWI might be affected by the hand used to heft the stimuli. Participants never saw the objects they hefted, removing all visual information about the stimuli. Once ready, the experimenter would initiate each trial which triggered a tone and immediately commenced the secondary task. The experimenter would then place the sphere in the participants hand shortly after the tone in each trial. This is important because it meant that the participant had already begun the secondary task by the time they experienced the objects’ weights. Thus, they did not have time to make a judgement about the object’s weight before their cognitive resources were allocated to the secondary task. Further, participants could not see the sphere, therefore they were unable to gauge the object’s weight until it was placed into their hand. They were asked to heft the sphere in the hand it was presented to for the duration of each trial. The order of stimulus presentation was randomised for each participant. This hefting task was largely the same between the familiarisation phase and experimental conditions. At the conclusion of a trial, the experimenter promptly removed the sphere before asking the participant for a perceptual magnitude estimate, which required participants to generate a numerical rating of perceived weight for the object [4, 10, 40]. These estimates were not confined to a pre-determined weight scale, such as grams, or an anchor. Instead, participants were asked to apply a scale of their choosing as long as they remained consistent to this scale throughout the study. It was stressed to participants that larger numbers should always correspond to heavier objects. Importantly, magnitude estimates were collected only after the sphere had been removed from the participant’s hand. This removed the ability for the participant to shift their focus back to the object when asked for an estimate.

### Secondary task

The secondary task required participants to respond with a foot-pedal press to visually presented stimuli. These stimuli changed in color and orientation, and participants had different instructions based on load condition. This task was built using parameters that were similar to previous dual-task studies [41, 42]. In the centre of the monitor, participants were presented with a cross subtending a visual angle of 1.1° in width and 1.5° in height on a black background (luminance of 6.4 cd/m^2^). In front of the cross, there was a grey (RGB: 128, 128, 128) fixation dot presented in Courier New font at a 30-point font size. The cross stimuli changed in both colour and orientation. The five colours included were red (RGB: 235, 11, 1), blue (RGB: 9, 173, 244), green (RGB: 132, 212, 65), yellow (RGB: 254, 253, 5), and pink (RGB: 255, 138, 150). Upright and inverted versions of each colour were used for a total of ten stimuli (Fig 1B). Crosses were presented in a random order in each trial, with each variation appearing three times per trial for a total of 30 presentations. To maintain consistency between conditions, each cross was displayed for 750 ms with a 100 ms fixation dot between each, totalling 25,500 ms per trial. Participants were required to respond to particular crosses by pressing the foot-pedals whilst they hefted a task object in one of their hands. Instructions for the secondary task differed depending on the experimental condition with the low-load task being easier than the high-load one (see below).

### Procedures

#### Familiarisation phase

Each participant completed a familiarisation phase after completing the pre-experiment questionnaires and vision checks. The purpose of this phase was to acquaint participants with giving weight estimates, the set-up, and hefting objects with their hands behind a curtain. This phase consisted of the hefting task outlined earlier with both spheres from the practice set only. Each stimulus was presented twice to each hand in randomised order for a total of eight trials. During the task, the same grey fixation dot described for the secondary task was displayed in the centre of the monitor. Participants were instructed to fixate on this dot at all times. No time limit was imposed for the familiarisation task. Participants were told to manually explore the sphere for the purposes of assessing its weight until they felt confident enough to provide a weight estimate.

#### Experimental conditions

Participants then completed the four experimental conditions in an order counterbalanced across participants. Each condition took roughly 15 minutes to complete. Every trial within a condition lasted 25,500 ms.

#### No-load

In this condition, participants completed the hefting task whilst fixating on the grey fixation point. An audible tone indicated the start and end of each trial. The experimenter placed a sphere from the experimental set into one of the participant’s hands following an audible tone. A second tone then signalled the end of the trial and the experimenter promptly removed the sphere. A diagram of the sequence of events in a trial can be seen in Fig 1C. This was repeated until the participant manipulated each sphere once in each hand for a total of 24 trials.

#### No-load with gloves

In this condition, procedures were identical to the no-load condition described above, with the exception being that participants now wore fleece gloves (Fig 2B) when manipulating the spheres.

#### Low-load

In this condition, participants completed the hefting task whilst simultaneously completing the secondary task. Participants were instructed to respond to the secondary task by pressing the right-foot pedal whenever the cross was red, regardless of its orientation. An instruction screen appeared between trials to remind participants of the required response for this condition. To facilitate understanding of the instructions, participants completed ten practice trials of the secondary task before doing the dual task. Following these practice trials, participants completed 24 real trials, which included both the hefting and secondary tasks performed at the same time.

#### High-load

Procedures for the high-load condition were identical to the low-load one with the following exception. Participants were instructed to press the left-foot pedal whenever the cross was green and inverted, and the right-foot pedal whenever the cross was yellow and upright. This made the condition more demanding than the low-load one.

#### Data preparation and analysis

Prior to analysis, the magnitude estimates were normalised to ensure that all participants’ estimates were on the same overall scale for analysis. Namely, all raw magnitude estimates were transformed into z-scores. This was accomplished by subtracting the mean of all magnitude estimates from each individual score and dividing this by the standard deviation of all estimates.

The analyses were carried out using IBM’s SPSS Statistics version 23 [43], GraphPad Prism version 7 (GraphPad Software, Inc.; La Jolla, California, USA), and JASP software version 0.8.1.2 (University of Amsterdam, Amsterdam, The Netherlands). Greenhouse-Geisser corrections were applied whenever the assumption of sphericity was not met according to a Mauchly’s sphericity test. Z-scores from the familiarisation task were examined with a 2 (Size: large, small) x 2 (Hand: left, right) ANOVA to investigate the effect of Size and Hand on the perceptual strength of the illusion. Z-score estimates from the experimental conditions were examined with a four-way repeated measures ANOVA. Factors included Condition (high-load vs low-load vs no-load vs no-load with gloves), Weight (161g vs 171g vs 181g vs 191g vs 201g vs 211g), Hand (left vs right), and Size (small vs large). Bonferroni-corrected, post-hoc pairwise comparisons were conducted to examine further any interactions and main effects found significant by the ANOVAs. Significance was established at an alpha level of .05 and all *p*-values reported were corrected for multiple comparisons. Finally, to confirm a difference in difficulty between high-load and low-load tasks, paired samples *t*-tests were conducted on accuracy and reaction time for both conditions, with both reaction time and accuracy being considered to account for potential speed-accuracy trade-offs [44]. Data obtained from practice trials in the main experiment were not analysed.

Null hypothesis statistical testing (NHST) only allows one to make judgements as to whether the alternative hypothesis can be accepted by rejecting the null hypothesis. It does not offer any information regarding the viability of the null hypothesis [45, 46]. We conducted Bayesian statistics to overcome this limitation. The use of NHST, effect sizes, and Bayesian statistics provide stronger and more compelling evidence for the presence as well as the absence of an effect when they converge. As recommended by Jeffreys [47], a Bayes Factor (*BF*_*10*_) greater than 3 was considered to provide substantial support for the alternative hypothesis while the inverse of this (*BF*_*10*_ < 0.33) was considered to provide substantial support for the null hypothesis.

## Results

### Summary

The statistical analyses presented in the subsequent sections reveal several key findings. Participants experienced a strong SWI in the familiarisation task. In the experimental conditions, participants rated the small objects as lighter when wearing gloves compared to when they did not. There were no differences observed for the large object in any condition. On the other hand, the strength of the illusion did not differ when participants performed the secondary task, regardless of load, compared to when they did not. No speed-accuracy trade-off effects were observed. The full dataset is available for download at: https://doi.org/10.26181/5c9c7450dd79a.

### Magnitude estimates of perceived weight in the familiarisation experiment

The ANOVA revealed a main effect of Size, *F*(1,24) = 1321.07, *p* < .001, $\eta_{p}^{2}$ = .98. Participants perceived the smaller sphere heavier than the larger one. No main effect was observed for Hand, *F*(1,24) = 1.14, *p* = .297, $\eta_{p}^{2}$ = .05, suggesting the illusion did not change based on the hand with which they hefted the object. No interaction effect was found between the two factors, *F*(1,24) = .01. *p* = .922, $\eta_{p}^{2}$ = 0. Bayesian paired samples t-tests indicate substantial evidence in favour of heavier estimates of weight for the smaller compared to the larger object (*BF*_*10*_ = 7,555). In contrast, no differences were demonstrated between the left (non-dominant) and right (dominant) hand (*BF*_*10*_ = .35). Taken together, the results from the familiarisation experiment demonstrate that participants perceived the smaller sphere as heavier than the larger one, regardless of the hand to which it was presented.

### Magnitude estimates of perceived weight in the main experiment

**Fig 3:**
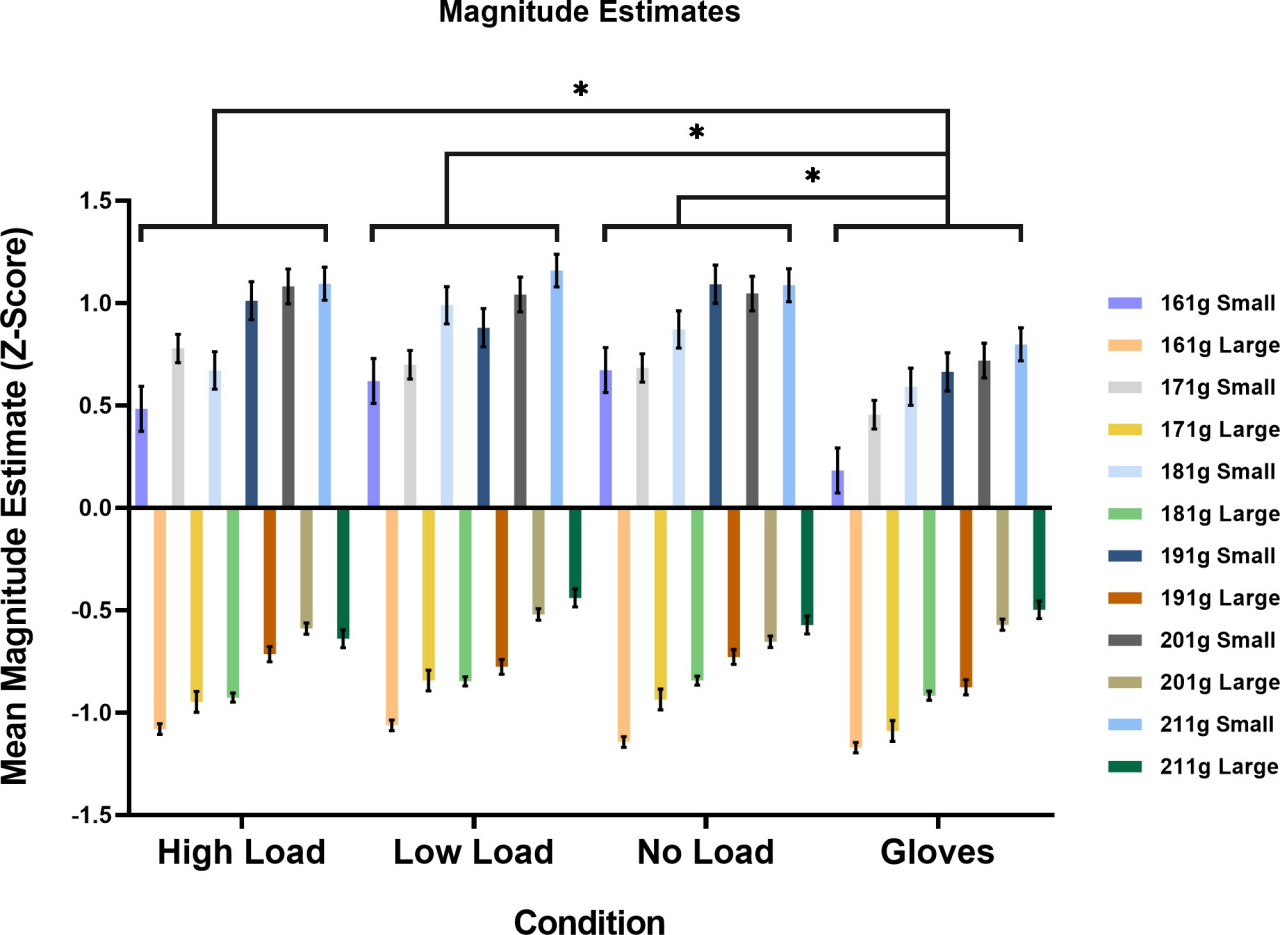
Results from the main experiment. Results from the ANOVA on weight estimates in the main experiment. The X-axis displays the four experimental conditions. The Y-axis shows the mean standardised weight estimate for each object weight and size. Larger scores indicate a higher weight estimate. Error bars represent means ± *standard errors around the mean (SEM)*. Asterisk (*) represent significant differences between the no-load glove condition and a different condition at *p* < .01 after corrections were applied from multiple comparisons using the Bonferroni method.

Analysis of estimates from the experimental conditions indicate lower estimates for small objects when wearing gloves. However, perceptual estimates were not impacted by the addition of cognitive load when completing a secondary task. See Fig 3 for a graphical representation of these results.

ANOVA revealed a Condition x Size interaction, *F*(3,72) = 5.60, *p* = .002, $\eta_{p}^{2}$ = .19. All other interactions were not significant (all *p* ≥ .05). All main effects were significant (*p* ≤ .022) except a main effect of Hand (*p* = .884). Post hoc analyses focused on delineating the significant two-way interaction. As demonstrated in Fig 3, estimates in the gloved condition depended on the size of the objects. More specifically, the Condition x Size interaction was driven by lower estimates provided for the smaller object when wearing gloves compared to all other conditions (all *p* < .001). Whereas estimates for the large object did not differ in any condition (all *p* ≥ .377). Bayesian paired-sample t-tests corroborate these results, indicating substantial evidence for the alternative hypothesis when investigating estimates given to the *small* objects. More specifically, lower estimates were observed when wearing gloves compared to the high-load (BF_10_ = 3.72), low-load (BF_10_ = 162.91), and no-load (BF_10_ = 400.95) conditions. Whereas substantial evidence was found for the null hypothesis when investigating estimates given to the *large* object when wearing gloves. Specifically, the gloves did not differ from the high-load (BF_10_ = 0.22), low-load (BF_10_ = 0.49), or no-load (BF_10_ = 0.24) conditions.

### Accuracy and reaction times on the secondary task

Participants were both slower and less accurate in the high-load compared to the low-load task (see Table 1). These findings confirm that the high-load task was more difficult and cognitively taxing. Specifically, participants were more accurate in the low-load relative to the high-load task, *t*(24) = 7.01, *p* < .001, *d* = 1.17, *BF*_*10*_ = 61,973. Participants were also slower to respond in the high-load than the low-load task, *t*(24) = 19.93, *p* < .001, *d* = 2.72, *BF*_*10*_ = 235,991.

**Table 1:** Reaction time and accuracy results from secondary task.

	Reaction Time (ms)		Accuracy (%)	
	*M*	*SD*	*M*	*SD*
High Load	629.01	45.96	0.76	0.16
Low Load	502.01	47.49	0.93	0.11

### Speed-accuracy trade-off

No evidence was found for the existence of a speed-accuracy trade-off. Instead, a negative as opposed to a positive correlation was found between response speed and accuracy, *r*(23) = -.76, *p* < .001. This indicates that the participants made more errors when they were slower not faster, which highlights further differences in task difficulty between the low-load and high-load tasks (see Discussion).

## Discussion

The present study determined the relative contribution of cognitive and sensory mechanisms in the SWI. This was done by varying the amounts of cognitive load in a dual-task and the quality of somatosensory feedback by wearing versus not wearing gloves. Overall, our results demonstrate that the illusion is more strongly driven by bottom-up sensory processes than top-down cognitive mechanisms.

The first key finding is that participants experienced an illusion of equal strength in the no-load, low-load, and high-load conditions. Namely, the perception of the illusion remained the same whether completing a secondary task or not, regardless of the degree of additional cognitive load. This finding falls in line with those reported by Trewartha and Flanagan [34], who also observed that cognitive load had little impact on the SWI. However, the current study’s findings extend from those described by Trewartha and Flanagan by demonstrating that cognitive load has little impact on illusory strength in the SWI when vision is unavailable and by providing more information on the validity of our dual task by reporting accuracy and reaction time measures on the secondary task. If the illusion relies heavily on cognitive mechanisms, then illusory strength should have weakened with an increase in cognitive load. Instead, we saw that illusory strength did not change as a function of cognitive load, which demonstrates that cognitive explanations may not be as crucial in driving the SWI as some postulate [10–12]. We can also confidently say that our dual-task was effective in taxing cognitive processing more in the high-load compared to the low-load conditions as evidenced by lower accuracy and longer reaction times in the secondary task in the former compared to the latter.

The second key finding is that illusory strength was weakened when the quality of somatosensory information about the stimuli was diminished with gloves. In the no-load with gloves condition, the strength of the SWI was reduced compared to the other conditions. However, this reduction was driven by changes in weight estimates for the small but not the large object. This effect on the small but not the large object is interesting because it suggests that the processing of size but not weight information was disrupted. Small and large objects had the same weight. If the processing of weight information was disrupted then we would expect changes in weight estimates for both objects. We know from tactile discrimination experiments that it is more difficult to differentiate between two points on the skin when they are close together than when they are further apart, particularly in patients with peripheral deafferentation [48]. In a similar manner, it could be the case that wearing gloves makes it more difficult to differentiate the size of objects, particularly when they are smaller. Regardless of the nature of the information that is being disrupted, this second key finding demonstrates that the illusion is driven strongly by low-level somatosensory processing. It is important to emphasise that participants had no visual information about the stimuli. Thus, the only difference between the two no-load conditions was the quality of somatosensory information about the objects. Therefore, our findings suggest that somatosensory information is particularly important in driving the illusion, at least when vision is removed. This is consistent with findings from the study by Ellis and Lederman [21] described earlier in the Introduction.

Further evidence for the importance of somatosensory processing was demonstrated in patient IW, who suffers from peripheral deafferentation [24]. Buckingham and colleagues [24] found that patient IW had accurate pre-lift expectations of the object weights according to their size (i.e., expecting larger objects to be heavier). However, he experienced no illusion when lifting these objects. These findings are consistent with the current study’s findings. It appears that somatosensory information, including tactile (touch) and kinaesthetic (awareness of body position and movements) information, are important for perceiving the SWI. Reducing the quality of this information (e.g., with gloves) and removing it altogether (e.g., patient IW or lifting with strings) weakens and eliminates the illusion, respectively. Next, we offer two explanations as to how a noisy size signal might diminish the SWI.

In a recent review of the SWI, Saccone and Chouinard [9] proposed that size can influence weight perception more strongly than other object features because of the speed with which the brain can process this information. In the somatosensory system, different types of information are transmitted and processed at different speeds. Proprioception is faster than touch, which is faster than either pain or temperature [49, 50]. Amazeen and colleagues demonstrated on several occasions the strong influence of size information obtained kinaesthetically on the SWI [51–53]. This information is processed by the fast proprioceptive channel, which perhaps fulfils a role similar to the fast magnocellular pathway in the visual system, which transmits and processes low-spatial frequency information, including information about the size of objects, pre-consciously in order to provide contextual information for influencing perception, which occurs later in time [54, 55]. Saccone and Chouinard’s theory [9] might explain why the size of obejcts influenced weight perception less strongly when this information was obtained less precisely in the condition where participants wore gloves. The contextual information regarding object size was noisy and therefore less effective in modulating weight perception.

Alternatively, a noisy size signal may reduce the brain’s ability to compute density, which some have argued or demonstrated is important for the SWI [17–19, 56–60]. Wolf et al. [19] performed a series of experiments to test this possibility. In one experiment, the authors had participants lift objects via strings, which eliminated somatosensory feedback about object size, and varied the degree of visual access to the objects, therefore varying the precision of size information. More precisely, they altered visual acuity by having their participants wear special goggles offering no, poor, moderate, or full visibility of the work space in front of them. Objects varied in size, mass, and density and participants provided weight estimates after each lift. Size and density had no effect on weight estimates in the viewing condition without visibility but had more influence on weight perception as visibility increased and participants experienced a stronger SWI. The authors then proceeded to fit their data to a model they devised where density predicted weight perception as a function of visibility. Their model explained more than 85% of the variance per individual (group median: ~94%), demonstrating that density and the abilitiy to compute it based on the quality of size information available to a person contributes substantially to the SWI. Wolf et al. [19] obtained similar results in a different experiment where they manipulated the quality of size information obtained haptically. This was achieved by having participants lift their objects using three different natural grip types (fully enclosed in the hands, a precision grip, or lifting via a string). This achieved three levels of haptic interaction with the objects–with an enclosed grip providing the most reliable information, precision grips being slightly less reliable, and strings to remove haptic information altogether. These three conditions contrast the current study’s methods in which we utilised an enclosed grip in one hand, but attempted to hinder the reliability of information with the gloves.

Nonetheless, there are a two methodological issues to consider. The first relates to timing. A recent paper by Plaisier and colleagues [61] demonstrates that perceptual judgements of weight are formed within the first 300 ms of lifting an object. If one were to assume that Plaisier and colleague’s [61] conclusions are correct then it could be the case that participants formed an intitial weight judgment in the beginning of each trial within the first 300 ms and then revised their judgements continuously throughout the remaining ~25 seconds. If this were indeed the case then it is remarkable that wearing gloves would still affect the strength of the illusion with all this time to revise weight judgements. Although there is always the possibility that providing less opportunities for participants to revise their weight judgements with shorter trials may have lead to effects of cognitive load, it is important to underscore that our procedures allow us to conclude that wearing gloves impedes the SWI to a greater extent than cognitive load when both exert an interference for the same amount of time–underscoring the relative importance of the former over the latter. Future work could consider re-examining the effects of cognitive load with shorter trials. The second relates to the nature of our secondary task. Namely, could a different secondary task requiring a different set of cognitive demands yield greater interference? This is certainly a possibility and requires further testing with different secondary tasks. Our secondary task increased mental workload and required participants to attend viligently to changes in the physical features of a stimulus, maintain rules in working memory, and make perceptual decisions. None of these extra demands affected the SWI.

## Conclusions

The current study determined the relative contributions of bottom-up sensory and top-down cognitive processes in the SWI. We demonstrate that cognitive load on a secondary task had no influence but that illusion strength was weaker when the quality of information about object size was diminished when participants wore gloves. Together, these findings demonstrate that the processing of somatosensory information exerts a stronger influence on the SWI than cognitive processing. We conclude that the illusion is more influenced by bottom-up sensory than top-down cognitive processes–at least when visual information about the objects is not provided.
